# A Deep-Learning Method for Radar Micro-Doppler Spectrogram Restoration

**DOI:** 10.3390/s20175007

**Published:** 2020-09-03

**Authors:** Yuan He, Xinyu Li, Runlong Li, Jianping Wang, Xiaojun Jing

**Affiliations:** 1 Key Laboratory of Trustworthy Distributed Computing and Service (BUPT), Beijing University of Posts and Telecommunications, Beijing 100876, China; yuanhe@bupt.edu.cn (Y.H.); lixinyu@bupt.edu.cn (X.L.); lirunlong@bupt.edu.cn (R.L.); jxiaojun@bupt.edu.cn (X.J.); 2 Faculty of Electrical Engineering, Mathematics and Computer Science (EEMCS), Delft University of Technology, 2628CD Delft, The Netherlands

**Keywords:** image restoration, radar micro-doppler spectrogram, fully convolutional network, generative adversarial network

## Abstract

Radio frequency interference, which makes it difficult to produce high-quality radar spectrograms, is a major issue for micro-Doppler-based human activity recognition (HAR). In this paper, we propose a deep-learning-based method to detect and cut out the interference in spectrograms. Then, we restore the spectrograms in the cut-out region. First, a fully convolutional neural network (FCN) is employed to detect and remove the interference. Then, a coarse-to-fine generative adversarial network (GAN) is proposed to restore the part of the spectrogram that is affected by the interferences. The simulated motion capture (MOCAP) spectrograms and the measured radar spectrograms with interference are used to verify the proposed method. Experimental results from both qualitative and quantitative perspectives show that the proposed method can mitigate the interference and restore high-quality radar spectrograms. Furthermore, the comparison experiments also demonstrate the efficiency of the proposed approach.

## 1. Introduction

Human target detection and activity recognition in radar are attracting more and more attention recently, and have been applied in many areas, such as assisted living [1] and health monitoring [2]. Compared with optical sensors and infrared sensors, radar has its unique advantages such as robustness to the environment, low-power, penetrability, and protecting visual privacy [3]. Micro-Doppler (MD) spectrograms are often used for human activity recognition due to the characteristic of MD effect [4,5,6,7,8]. The MD effect refers to the additional frequency modulations on the returned radar signals, which are induced by the rotation, vibration, and other motions of human limbs. Since the motion patterns of different human activities are diverse, the micro-Doppler frequencies in the returned radar signals are activity-specific. As a result, the time-varying MD frequencies in spectrograms are vital to classify human activities.

However, in the real world, there is often interference that impacts the quality of the radar spectrograms, making the performance of the models for activity recognition drop significantly. In such circumstances, how to mitigate the interference and restore clear radar spectrograms becomes an essential topic to be investigated. Many anti-interference methods employing digital signal processing techniques have been proposed [9,10,11]. However, these methods mainly focus on the interference mitigation task. In addition, the radar signals that are impacted by the interferences cannot be recovered well, especially those disturbed by the interferences with long time duration or large frequency bandwidth.

Furthermore, deep-learning (DL) technique has been used for interference mitigation. Huang et al. [12] proposed a generative adversarial network (GAN) to remove the noise in radar spectrograms. This method shows the possibility of combining traditional methods with deep-learning approaches. However, this method may affect parts of the spectrogram without interference and introduce new uncertainties. Fan et al. [13] proposed a deep residual network for narrow-band interference and wide-band interference mitigation. Ristea et al. [14] proposed fully convolutional neural networks (FCNs) to remove the interference in the FMCW signals and outputs the corresponding clean range profiles. In this approach, the main goal is to mitigate the interference, but how to restore the interfered signals with good quality is not considered.

However, the main goal of these methods is to mitigate the interference in the radar images. How to restore the part of radar signals that are influenced by interference was not considered. To eliminate interferences, some useful radar signals are discarded during the interference mitigation process. In this paper, we propose a deep-learning method for both interference mitigation and radar spectrogram restoration. In contrast to the previous interference mitigation work, we integrate the interference mitigation and spectrogram restoration together and try to minimize the impact of interference cancellation on the spectrogram. It is noted that the proposed method can restore the spectrogram where the interference has long time span or long frequency span with sufficient accuracy.

Specifically, the proposed DL method is composed of two parts: an FCN for interference mitigation and a coarse-to-fine GAN for spectrogram restoration. We use the FCN as the first step to distinguish the interference part from the spectrogram. In addition, there is localization information in the mask produced by the FCN. Subsequently, a coarse-to-fine GAN is adopted for the further spectrogram restoration. The spectrogram, together with its mask, is then fed into the GAN for restoration. With the information of interference localization, the part of the spectrogram that is contaminated by the interference can be restored with better quality. Experimental results demonstrate from both qualitative and quantitative perspectives that the proposed DL model is effective in mitigating the interference and in obtaining a high-quality spectrogram.

The remainder of this letter is organized as follows. Section 2 describes the proposed DL-based spectrogram restoration method. Section 3 introduces the simulated radar dataset, the measured radar dataset, and the detailed experimental implementation. Section 4 presents the experimental results of the proposed method for interference mitigation and spectrogram restoration. Conclusions are drawn in Section 5.

## 2. Radar Spectrogram Restoration Using Deep Learning

### 2.1. Algorithm Overview

**Figure 1:**
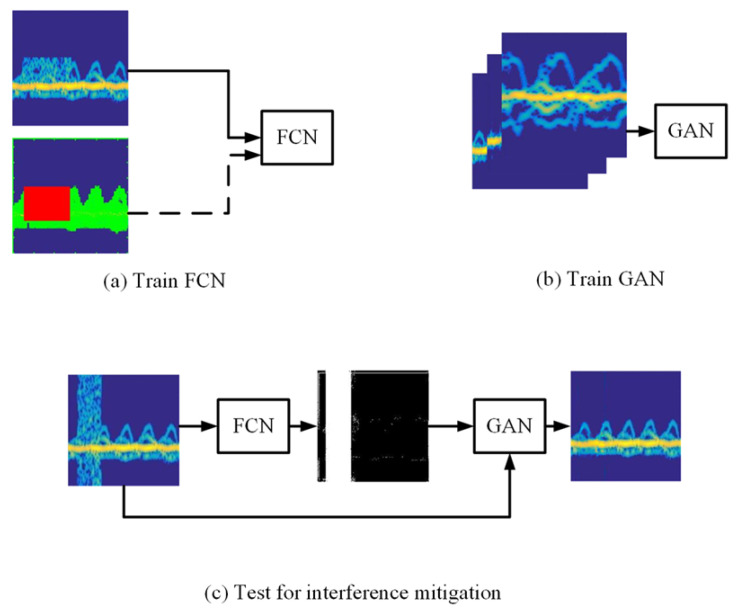
The pipeline of the proposed deep-learning method for interference restoration. (**a**) The FCN is trained with the spectrograms where there is interference with the supervision of the label. Then when a spectrogram with interference is fed into the trained FCN, the FCN can locate the interference accurately. (**b**) The GAN is trained with clear radar spectrograms. In this way, the GAN can learn the data distribution of clear spectrograms for further interference mitigation. (**c**) Finally, when a spectrogram with interference is fed into the FCN and fed into the GAN subsequently, a clear spectrogram can be restored.

In this section, we provide details of the proposed DL method in which we aim to restore a clear radar spectrogram by using FCN for interference localization and GAN for interference mitigation, respectively. The overall pipeline of the proposed method is illustrated in Figure 1. Specifically, the FCN is trained with the spectrograms where there is interference with the supervision of the label. Then, when a spectrogram **X** ∈$R^{m\times n}$ with interference is fed into the trained FCN. In this way, a mask **M**∈$R^{m\times n}$ that shows the localization of the interference on the spectrogram is produced by FCN. Next, as shown in Figure 1a, the GAN is trained with clear radar spectrograms to learn the data distribution of clear spectrograms for further interference mitigation. By taking advantage of the mask **M**, GAN is able to remove the interference accurately, and a clear spectrogram $X^{{}^{\prime }}$ ∈ $R^{m\times n}$ is restored. Finally, when a spectrogram with interference is fed into the FCN and fed into the GAN subsequently, a clear spectrogram can be restored. The proposed method that combines interference localization and mitigation together can make the spectrogram restoration more accurate and effective.

### 2.2. Fully Convolutional Network for Interference Localization

Fully convolutional networks have achieved great success for image segmentation task in many areas such as natural image processing [15,16], medical diagnosis [17,18] and radar target recognition [19,20]. The FCN is an extension of the classical convolutional neural network, and the main idea is to learn a mapping from input pixels to output pixels. It is a deep end-to-end model, which is composed of convolutional, pooling and upsampling layers.

We use VGG-19 [21] as the backbone of our FCN in this paper. The FCN performs a pixel-wise classification and classifies every pixel of the input image into one of the three categories (signal, background and interference). Cross entropy loss is adopted, and the loss function of the FCN is formulated as follows,

(1) $$L=-\frac{1}{N}\sum_{n=1}^{N}\sum_{c=1}^{M}y_{c}^{n}log\left(p_{c}^{n}\right)$$

where *M* is the number of categories and *N* is the number of pixels in an input spectrogram. $y_{c}^{n}$ is the one-hot label of pixel *n* while $p_{c}^{n}$ is the predicted result of the FCN for pixel *n*.

In this way, the interference region on the input spectrogram can be identified by the network, and a mask that is a three-value intensity image and has the same size as the input spectrogram is acquired. Furthermore, the localization information produced by FCN is used as the prior information of the interference, which can make the subsequent interference mitigation process more accurate and targeted.

### 2.3. Generative Adversarial Network for Spectrogram Restoration

**Figure 2:**
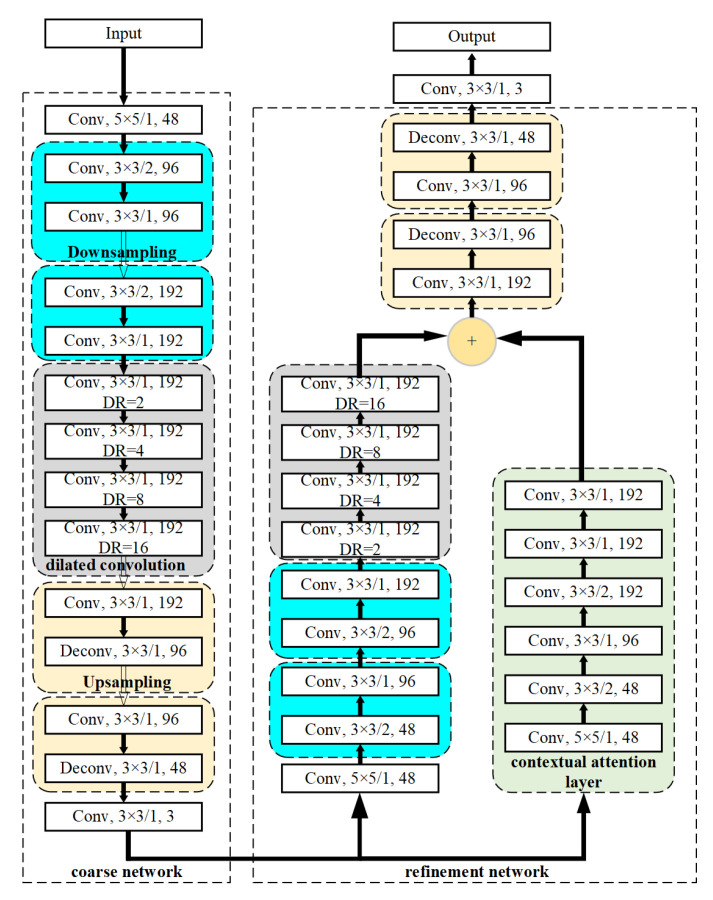
The structure of generator in the proposed GAN for interference mitigation. The descriptions with a form of “A × B/C, D” represent that there are *D* convolution kernels with a size of *A* × *B*. In addition, the convolution stride is *C*. DR refers to dilated rate of dilated convolution.

**Figure 3:**
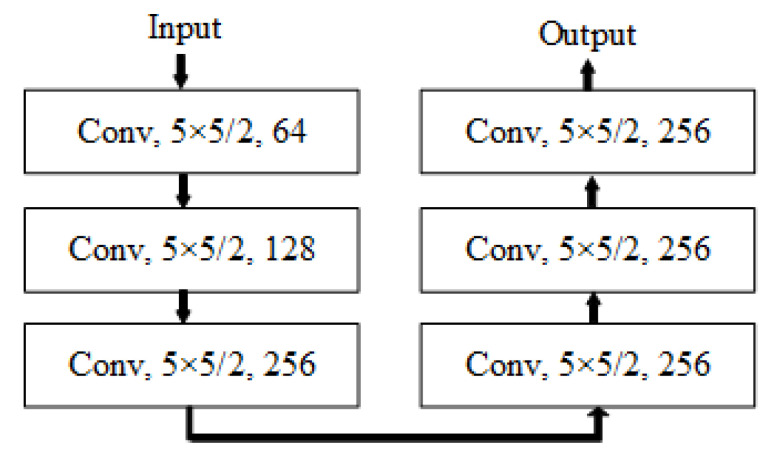
The structure of discriminator in the proposed GAN for interference mitigation. The descriptions with a form of “A × B/C, D” represent that there are *D* convolution kernels with a size of *A* × *B*. In addition, the convolution stride is *C*. DR refers to dilated rate of dilated convolution.

After locating the interference, the coarse-to-fine GAN [22] with a contextual attention scheme is adopted to remove interference and restore clear spectrograms. The architecture of generator in the GAN, as shown in Figure 2, can be roughly divided into two parts: coarse network and refinement network. A spectrogram with interferences and a binary mask that is output by the FCN model to locate the interferences are input to the GAN in pairs. Then, the coarse network makes an initial coarse restoration of the disturbed area of the spectrogram, and the refinement network takes the coarse prediction as inputs and makes further refined restoration. The structure of discriminator in the GAN is shown in Figure 3. A discounted reconstruction $\ell_{1}$ loss [22] is used for training the coarse network, and the refinement network is trained with the reconstruction loss as well as a modified WGAN-AP loss [23]. At the same time, the discriminator is also trained with the modified WGAN-AP loss. Furthermore, the discriminator and the refinement network are trained alternatively with this GAN loss [24]. WGAN uses the Earth-Mover distance [25]$W(P_{r},P_{g})$ to make the generated data distribution similar to the real data distribution.

(2) $$W\left(P_{r},P_{g}\right)=\underset{\gamma ∼\Pi (P_{r},P_{g})}{\inf}E_{(x,y)∼\gamma }[||x-y||]$$

where $\left(P_{r},P_{g}\right)$ denotes the set of all joint distributions γ(x,y) whose marginals are $P_{r}$ and $P_{g}$, respectively. E represents the expectation operation, and ||·|| represents $\ell_{1}$ norm. It is noted that both the reconstruction loss and the GAN loss optimize the model based on pixel-wise $\ell_{1}$ distances, which makes the coarse-to-fine GAN model trained easier and makes the optimization process stabler.

The objective function of the adversarial WGAN loss, which is constructed with the *Kantorovich-Rubinstein* duality, is formulated as follows,

(3) $$\min_{G}\max_{D\in D}E_{x∼P_{r}}\left[D\left(x\right)\right]-E_{\hat{x}∼P_{g}}\left[D\left(\hat{x}\right)\right]$$

where D is the set of 1-Lipschitz functions. *D(x)* and *D($\hat{x}$)* represent the output distributions of the real sample *x* and the generated sample $\hat{x}$. $P_{g}$ is the model distribution implicitly defined by $\hat{x}=G\left(z\right)$. z is the input to the generator *G*. Furthermore, a gradient penalty term [23] is added to the WGAN loss to form the loss function of WGAN-AP, which is formulated as

(4) $$\lambda E_{\tilde{x}∼P_{\tilde{x}}}\left(|\right|\nabla_{\tilde{x}}D\left(\tilde{x}\right){\left|\right|}_{2}{-1)}^{2}$$

where $\tilde{x}=\left(1-t\right)x+t\hat{x}$ is sampled from the straight line between points *x* and $\hat{x}$ sampled from distribution $P_{r}$ and $P_{g}$, respectively, and *t* is sampled from a normal distribution U∼[0,1]. In this paper, since we only predict hole regions, the gradient penalty is applied only to pixels inside the holes. As a result, the penalty term is reformulated as follows,

(5) $$\lambda E_{\tilde{x}∼P_{\tilde{x}}}\left(|\right|\nabla_{\tilde{x}}D\left(\tilde{x}\right)⊙\left(1-m\right){\left|\right|}_{2}{-1)}^{2}$$

where *m* represents the input mask, as shown in Figure 1c, and the mask value is 0 for missing pixels and 1 for elsewhere. λ is set to 1 in the experiments.

**Figure 4:**
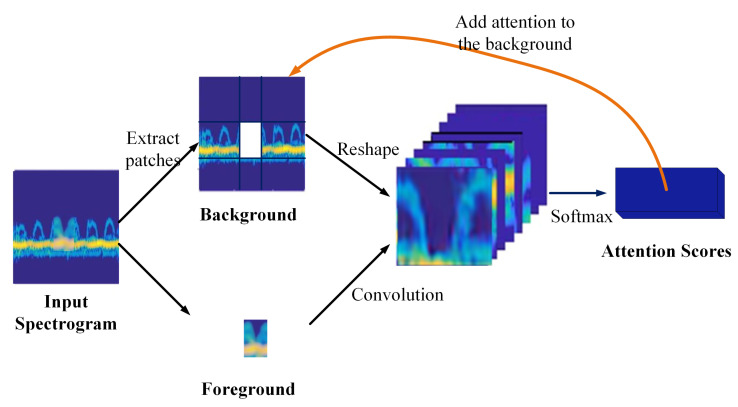
Contextual attention layer. Several patches (3 × 3) are extracted from the background and reshaped to the size of the missing part feature maps after two downsampling blocks.

Furthermore, since convolutional neural networks use local convolutional kernels to process input data, it is not effective to learn semantic information from distant spatial locations. As a result, a contextual attention layer is proposed, as shown in Figure 4. It can learn where to extract feature information from the clean part (background) of the spectrogram to restore the disturbed part (foreground). Specifically, several patches (3 × 3) are first extracted from the background and are reshaped to the size of the foreground patch after two downsampling blocks. To measure the similarities between the foreground patch with the background ones; the normalized inner product is used between foreground patch *m* with background patches *n*,

(6) $$S=\langle \frac{m}{\left|m\right|},\frac{n}{\left|n\right|}\rangle$$

where *S* indicates the similarity between *m* and *n*. Then, a scaled *softmax* is used to get the attention score for each pixel in the background patch *n* with $S^{*}$ = *softmax*(λ, *S*), and λ is a constant value. In this way, the attention scores of the whole background are obtained. Finally, the weighted background patches are used to reconstruct foregrounds.

## 3. Experiment Implementation

### 3.1. Simulated Radar Dataset

Most deep-learning algorithms need a large amount of data for training, but it is very difficult to collect measured data without noise and interference. So we use the Motion Capture database (MOCAP) [26] from Carnegie Mellon University (CMU) to simulate a micro-Doppler spectrogram dataset. MOCAP provides 2605 trials of human activities in 6 categories and 23 subcategories. Moreover, the captured human motion skeleton consists of 31 joint points. In this paper, we use six joint points, including the left hand, right hand, thorax, head, left foot, right foot, and five motions, including walking, running, jumping, boxing and standing.

The center frequency of radar used in simulation is 1.7 GHz, and bandwidth is 800 MHz. This experiment simulates a person moving towards the radar. The sampling frequency is 3 GHz. The received radar data are divided into several segments of 1 s. In addition, the overlap between adjacent segments is 0.9 s. Then a 1024-point Short-Time Fourier Transform (STFT) is used to process the data segments as follows.

(7) STFT(t,f)=∫x(t+τ)g(τ)exp(−j2τ)dτ

where x(t) is the received signal, g(t) is a sliding window function (e.g., a Hamming window), *t* is time, and *f* is frequency. Furthermore, to simulate the interference in the simulated radar spectrograms, we use additive white Gaussian noise (AWGN) with different time span and frequency span as interference and add them to spectrograms.

### 3.2. Measured Radar Dataset

The measured radar data are collected with a UWB radar module named PulsON 440. The center frequency is 4.0 GHz, and the pulse bandwidth is 1.8 GHz. The experiment is performed in an indoor environment. The radar is placed at the height of 1 m, and activities are performed in the line of sight of the radar. The measurement range of the radar is between 1.5 m and 7.5 m. The motion data of the following five activities are collected: (a) directly walking towards/away from the radar (walking); (b) boxing while standing in place (boxing); (c) directly running towards/away from the radar (running); (d) jumping forward (jumping); and (e) running in a circle (circle running).

Since it is difficult to collect a measured dataset with specific interference, we add AWGN to a random section of the origin radar spectrograms to simulate the accidental interference during the data acquisition.

### 3.3. Measure Metrics

To evaluate the anti-interference situation, we use Peak Signal-to-Noise Ratio (PSNR) and Structural Similarity (SSIM) as evaluation metrics [27]. PSNR is calculated by inputting the mean square error (MSE) of two input images. It shows the gap between the pixels of two images. SSIM focuses more on the similarity between structure and contrast of images.

(8) $$MSE=\frac{1}{n^{2}}\sum_{i=0}^{n-1}\sum_{j=0}^{n-1}{\left[x\left(i,j\right)-y\left(i,j\right)\right]}^{2}$$

(9) $$PSNR=10\log_{10}\left(\frac{{Max}^{2}}{MSE}\right)$$

(10) $$SSIM\left(x,y\right)=\frac{\left(2\mu_{x}\mu_{y}+c_{1}\right)\left(2\sigma_{xy}+c_{2}\right)}{\left(\mu_{x}^{2}+\mu_{y}^{2}+c_{1}\right)\left(\sigma_{x}^{2}+\sigma_{y}^{2}+c_{2}\right)}$$

where Max is the maximum value a pixel can take, $\mu_{x}$ is the average value, $\sigma_{x}^{2}$ is the variance, $\sigma_{xy}$ is the covariance, and *c* is a constant.

### 3.4. Training Details

For simulated radar data, the short-time Fourier transform (STFT) is performed first. A time window of 0.1 s is adopted with an overlap of 0.09 s. Then, the spectrogram, which is defined as the square modulus of the STFT with normalization, is obtained. For measured radar data, the moving target indicator (MTI) is adopted to remove the background clutter. Then, the measured spectrograms are obtained with the same process of simulated spectrograms.

The micro-Doppler spectrograms with a size of 256 × 256 without interference is shown in Figure 3. During training, we use ImageNet [28] to pre-train GAN to reduce training time and improve training effect. The parameters of FCN is initialized with those of the pretrained VGG-19. There are approximately 1000 pictures prepared for training, 80% of which is used for training while the others for testing. During testing, the mask produced by FCN is input to the GAN together with the masked image.

All experiments are implemented on TensorFlow [29] v1.3, CUDA V8.0, with GPU GTX1080TI. Batch size is set to 16; the learning rate is set to 0.0005 and 0.0001 for the GAN and FCN, respectively. After the proposed hybrid FCN and GAN model is trained, we use the trained model on a test dataset to obtain the test results and verify the performance of the proposed method. To simulate the radar signals with diverse interferences, we randomly set the values of SNR, interference duration, interference bandwidth, and interference intensity. Then, 100 interferences are simulated and added to clear radar signals to form a test radar dataset. When we have *m* radar spectrograms and add the 100 interferences on each of the spectrograms, 100 × *m* different spectrograms with interferences are obtained. Next, we test the trained hybrid model on the test data, and the statistical average values of PSNR and SSIM can be obtained and shown in the experimental results for further analysis, respectively.

## 4. Experimental Results

In this section, we conduct interference mitigation and spectrogram restoration experiments with both simulated and measured data to demonstrate the effectiveness of the proposed method. Qualitative and quantitative evaluations are adopted. Moreover, to demonstrate the efficiency of the proposed method, we compare its performance with several typical interference mitigation and spectrogram restoration methods. The details of the methods for comparison are presented below.

- *Zeroing* [11], which is a simple and well-known approach, is treated as a baseline during the experiments. It performs interference mitigation by simply setting the time domain samples of interference to a value of zero. The prior information on the position of the interference in the time domain is known.
- *FCNs* [14] uses FCNs to remove the interference in the FMCW signals and outputs the corresponding clean range profiles. In this approach, the main goal is to mitigate the interference, and how to restore the interfered signals with good quality is not considered.
- *ResNet* [13] adopts the deep residual network (ResNet) for interference mitigation in synthetic aperture radar (SAR). In detail, an interference detection network and an interference mitigation network are proposed respectively to remove interference and restore clean SAR images.

### 4.1. Results of the Simulated Data

#### 4.1.1. Qualitative Evaluation

**Figure 5:**
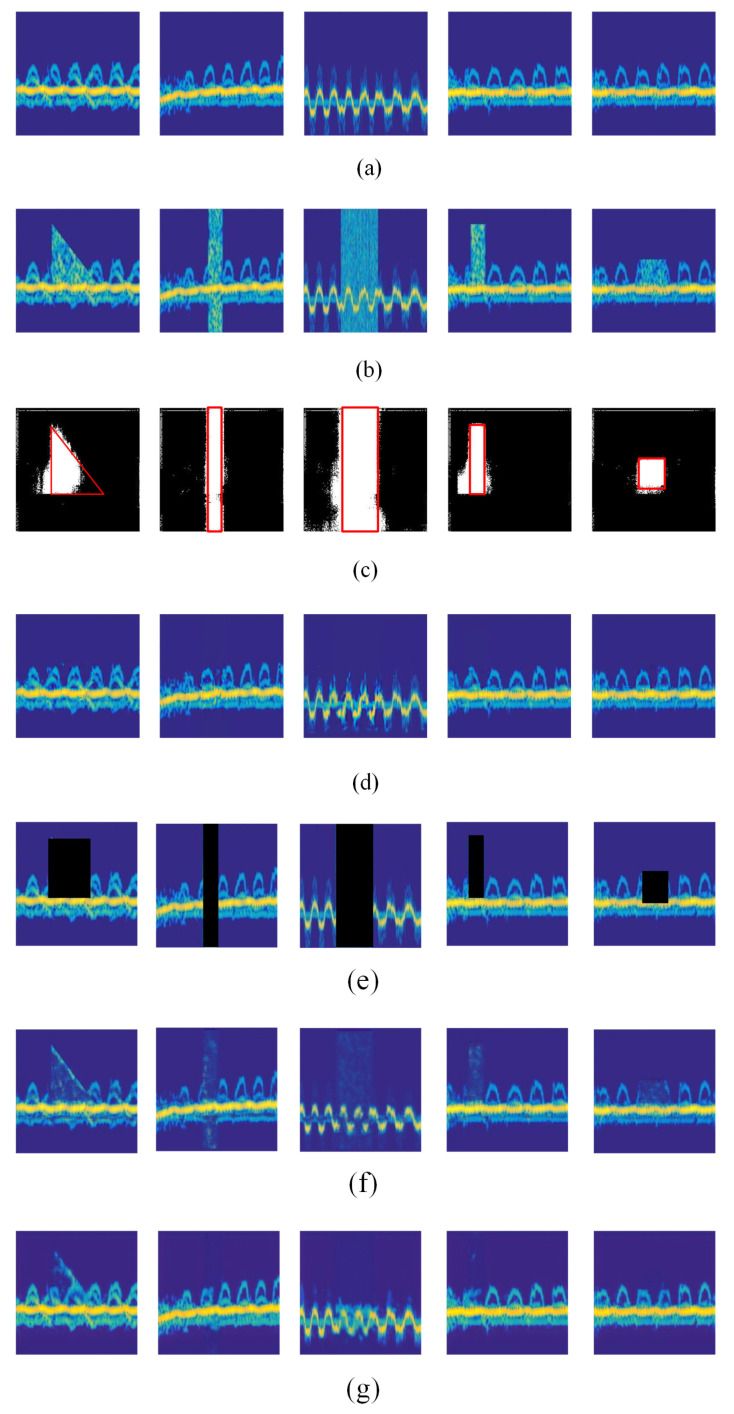
Results of the proposed method and three state-of-the-art methods on the simulated data. (**a**) The clean radar spectrograms. (**b**) The spectrograms with diverse interference. (**c**) The locations of interference detected by the proposed FCN model. The red boxes represent the ground truth of the locations of interferences. (**d**) The radar spectrograms restored with our method. (**e**) The spectrograms restored with *Zeroing*. (**f**) The spectrograms restored with *FCNs*. (**g**) The spectrograms with restored *ResNet*.

The qualitative performance of the proposed hybrid FCN and GAN model and the other three interference mitigation methods is shown in Figure 5. The clear simulated radar spectrograms are shown in Figure 5a, and the simulated spectrograms with diverse interferences are shown in Figure 5b. From Figure 5c, we can find that the proposed FCN-based interference mitigation method is able to accurately locate the position of the interferences and move them. Furthermore, it can be seen from Figure 5d that the proposed coarse-to-fine GAN can restore the part of the spectrogram that is impacted by the interference. It can be seen that the spectrograms are restored with good results, and are highly similar to the clean radar spectrograms, demonstrating that the proposed GAN for radar spectrogram restoration with good performance. Figure 5e–g shows the restored spectrograms using the methods *Zeroing*, *FCNs* and *ResNet*. As shown in this figure, the method *Zeroing* removes not only the interference but also the parts of spectrograms that are impacted by the interferences. Moreover, the methods *FCNs* and *ResNet* cannot remove the interference clearly. In addition, compared with *FCNs*, *ResNet* restores the interfered spectrograms with better performance.

#### 4.1.2. Quantitative Evaluation

Furthermore, the performance comparison from the quantitative perspective is conducted to verify the efficiency of the proposed hybrid FCN and GAN model. And the results are listed in Table 1. As shown in this table, the proposed GAN-based approach achieves the best performance among the four interference mitigation methods. In particular, a PSNR of 65.714 has been achieved, demonstrating that the proposed method is able to accurately detect the interference and remove the interference as much as possible. Moreover, the highest SSIM of 0.930 is also obtained by the GAN-based approach, indicating that the approach has good performance on restoring radar spectrograms and reconstructing the interfered time-frequency information. Additionally, followed by the method *FCNs*, *ResNet* achieved the second-best performance with an PSNR of 63.364 and a SSIM of 0.926. The two quantitative results are consistent with the qualitative results shown in Figure 5. Finally, the method *Zeroing* achieved the worst performance with an PSNR of 35.210 and a SSIM of 0.720. It is mainly because it performs interference mitigation by simply setting the time domain samples of interference to a value of zero. In this way, not only the interferences are mitigated, the radar signals are removed.

### 4.2. Results of the Measured Data

#### 4.2.1. Qualitative Evaluation

**Figure 6:**
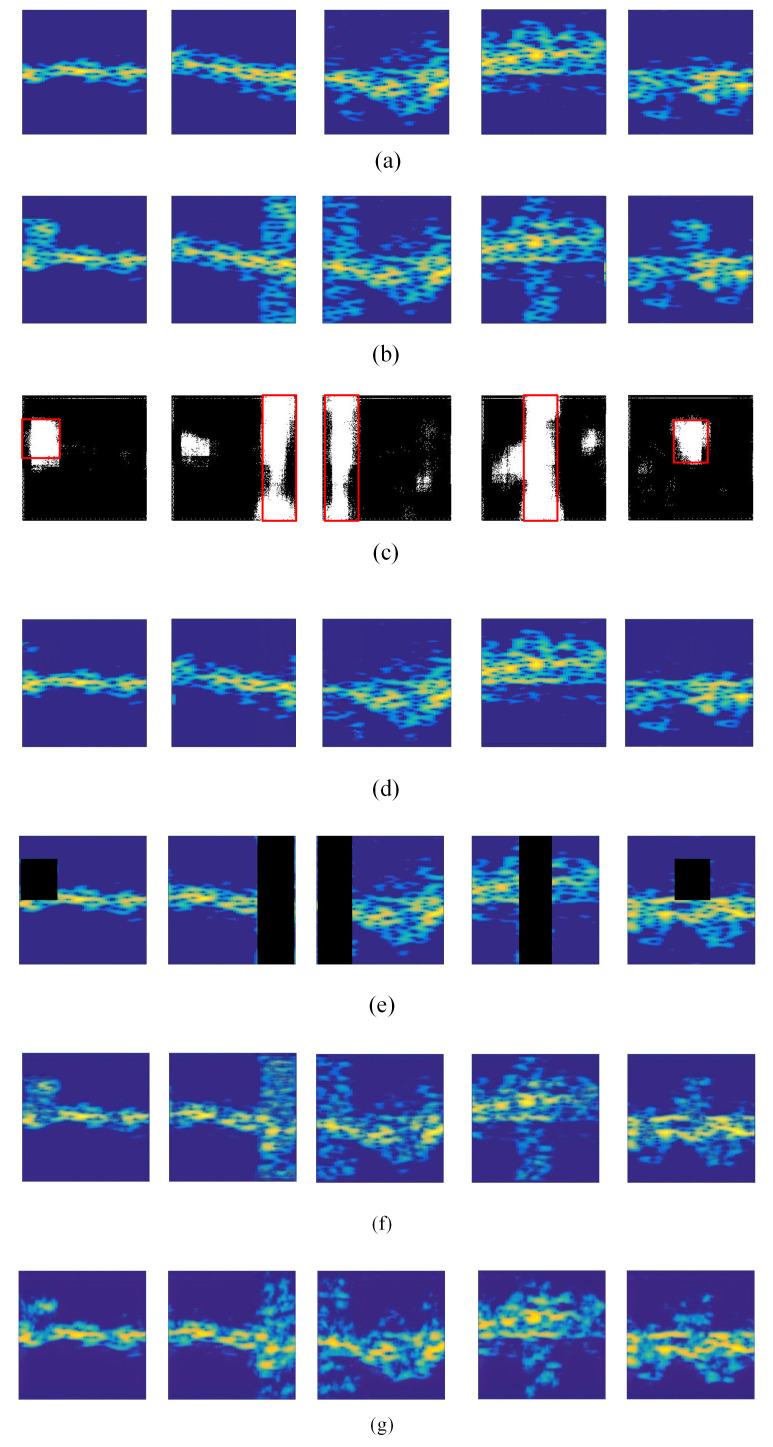
Results of the proposed hybrid FCN and GAN method and three state-of-the-art methods on the measured data. (**a**) The clean radar spectrograms. (**b**) The spectrograms with diverse types of interferences. (**c**) The locations of interferences detected by the proposed FCN model. The red boxes represent the ground truth of the locations of interferences. (**d**) The radar spectrograms restored with our method. (**e**) The spectrograms restored with *Zeroing*. (**f**) The spectrograms restored with *FCNs*. (**g**) The spectrograms with restored *ResNet*.

Figure 6 shows the results of measured radar spectrograms for interference mitigation and spectrogram restoration with our hybrid FCN and GAN method and the other three state-of-the-art methods. The clear measured radar spectrograms are shown in Figure 6a, and the corresponding spectrograms with diverse interferences are shown in Figure 6b. As shown in Figure 6c,d, the proposed method can locate the interference and restore the spectrograms with acceptable performance. Furthermore, though the FCN sometimes removes only the interference but also some original micro-Doppler frequency components, the GAN can restore the removed frequency components, which makes up for the weakness of the FCN model. As a result, with the collaboration of the FCN and the GAN, the interference is removed, and the interfered spectrograms are restored. Figure 6e–g shows the results of the three compared methods on the measured data. Similar performance to that on the simulated radar spectrograms is achieved. Compared with *FCNs*, *ResNet* can remove more interference while *FCNs* can restore more information of the interfered spectrograms. However, from the qualitative perspective, the performance of all three methods is worse than our DL method.

#### 4.2.2. Quantitative Evaluation

Furthermore, the quantitative evaluation in the performance of the three compared methods and the proposed hybrid FCN and GAN model is conducted with the measured radar data. The performance comparison results are listed in Table 2. It can be seen that the proposed method achieves the highest PSNR and the best SSIM. In particular, the average PSNR and SSIM of the restored spectrograms with *Zeroing* are both the lowest, followed by those of the spectrograms with *FCNs*. In addition, the average PSNR of spectrograms with *ResNet* is 51.249, and the average SSIM is 0.822. The highest PSNR of 51.724 and SSIM of 0.864 demonstrate that the proposed method can remove most of the interference while restoring the original spectrograms to the utmost. Additionally, the method *FCNs* and the method *ResNet* achieved similar performance for interference mitigation and spectrogram restoration. Furthermore, the same as the quantitative performance on the simulated radar spectrograms, the performance of the method *Zeroing* is the worst with a PSNR of 39.053 and a SSIM of 0.767.

In particular, we further analyze the performance of four methods (*Zeroing*, *FCNs*, *ResNet* and the proposed hybrid FCN and GAN model) on the measured radar spectrograms by calculating the PSNRs and SSIMs of the spectrograms corresponding to ’walking’, ’running’, ’jumping’, ’boxing’ and ’circle running’. The PSNRs and SSIMs are listed in Table 3. It can be seen that our method achieved the best performance for the activities ’jumping’, ’boxing’, and ’circle running’. As for ’walking’ and ’running’, the PSNR of spectrograms with the *FCNs* method is the highest, though our method obtains the highest SSIM. It may be because the *FCNs* method focuses more on interference mitigation instead of spectrogram restoration. As a result, the PSNR is high since the interference is mitigated to the utmost. However, how to restore the parts of the spectrograms that are contaminated by the interferences is not well considered in the *FCNs* method. In contrast, in our proposed method, the GAN part mainly focuses on spectrogram restoration, which makes a high SSIM possible. Furthermore, as shown in Table 3, the PSNR and SSIM of *FCNs* on the spectrograms corresponding to ’walking’, ’running’, ’jumping’ and ’boxing’ are higher than those of *ResNet*. In contrast, the PSNR and SSIM of *ResNet* on the spectrograms corresponding to ’circle running’ are higher than those of *FCNs*. However, as shown in Table 3, the average performance of *ResNet* on the whole spectrogram dataset is better than that of *FCNs*.

### 4.3. Performance Comparison on the Human Activity Recognition Task

In this subsection, to demonstrate the good performance of the proposed method for spectrogram restoration, we further conduct several task-specific experiments with the restored spectrograms for HAR. In detail, we first train *AlexNet* [30], which is a typical deep-learning model for classification, with clean simulated/measured radar spectrograms. Then, the restored simulated/measured radar spectrograms are fed into the trained *AlexNet* for classification. The classification accuracies of the spectrograms restored with *Zeroing*, *FCNs* and *ResNet* and our hybrid FCN and GAN model are listed in Table 4. As shown in this table, the restored spectrograms with the proposed method can be classified with the highest accuracies of 0.947 on the simulated data and 0.915 on the measured data. Hence, beyond the good performance from a quantitative and qualitative perspective, the performance for the activity classification task also indicates our method is able to remove the interference while retaining the valuable information in the original clean spectrograms. On the contrary, the experimental results of *Zeroing*, *FCNs*, and *ResNet* show that some vital information for activity classification is missing during the interference mitigation process, which degrades the classification performance.

## 5. Conclusions

In this paper, we propose a deep-learning-based model for interference mitigation and spectrogram restoration. In contrast to the previous interference mitigation approaches, the proposed method integrates the interference mitigation and spectrogram restoration tasks together, and try to minimize the impact of interference cancellation on the spectrograms. Specifically, the proposed method is composed of an FCN and a GAN. The former is used to mitigate inferences, and the latter is used to restore the parts of spectrograms that is disturbed by the interferences.

Several experiments with both the simulated and the measured radar spectrograms were performed to verify the effectiveness of the method. Experimental results show that the proposed method can restore high-quality radar spectrograms, with higher PSNR and SSIM when compared with the original interfered spectrograms. Furthermore, the comparison experiments with several interference mitigation methods demonstrate the superiority of the proposed approach.

In the near future, we will carry out research from the following aspects. First, since the proposed deep-learning model is not an end-to-end network, we will propose other deep-learning networks that can integrate the interference mitigation and the spectrogram restoration tasks together. Additionally, we will try to propose a general method that can mitigate the interference from different radar signal dimensions, such as 1-dimensional HRPP, 2-dimensional time-range domain, and 3-dimensional time-range-Doppler domain. Furthermore, we will explore more on how to mitigate the interferences of different signal types, such as LFM signal and communication signal.

## Figures and Tables

**Table 1:** Performance Comparison with the Simulated Radar Data.

	*Zeroing*	*FCNs*	*ResNet*	Ours
PSNR	35.210	58.935	63.364	**65.714**
SSIM	0.720	0.887	0.926	**0.930**

**Table 2:** Performance Comparison with the Measured Radar Data.

	*Zeroing*	*FCNs*	*ResNet*	Ours
PSNR	39.053	50.273	51.249	**51.714**
SSIM	0.767	0.812	0.822	**0.864**

**Table 3:** Performance on the Measured Radar Spectrograms of Five Human Activities.

		*Zeroing*	*FCNs*	*ResNet*	Ours
Walking	PSNR	48.557	**57.397**	56.465	56.805
	SSIM	0.883	0.879	0.850	**0.906**
Running	PSNR	33.087	**49.025**	48.676	47.279
	SSIM	0.687	0.769	0.758	**0.808**
Jumping	PSNR	34.904	47.947	46.741	**49.039**
	SSIM	0.695	0.772	0.757	**0.836**
Boxing	PSNR	45.541	55.961	54.483	**56.179**
	SSIM	0.886	0.908	0.903	**0.912**
Circle Running	PSNR	33.177	45.000	45.917	**49.267**
	SSIM	0.685	0.784	0.794	**0.856**

**Table 4:** Performance Comparison for Human Activity Recognition with the Restored Spectrograms.

	*Zeroing*	*FCNs*	*ResNet*	Ours
Simulated Data	0.855	0.864	0.866	**0.947**
Measured Data	0.805	0.821	0.819	**0.915**

## Abbreviations

The following abbreviations are used in this manuscript:

HAR	Human Activity Recognition
MOCAP	Motion Capture
MD	Micro-Doppler
IMAT	Iterative Method with Adaptive Thresholding
RFmin	Ramp Filtering
DL	Deep-Learning
GRU	Gated Recurrent Unit
CS	Chirp Sequence
GAN	Generative Adversarial Network
AWGN	Additive White Gaussian Noise
PSNR	Peak Signal-to-Noise Ratio
SSIM	Structural Similarity
MSE	Mean Square Error
STFT	Short-Time Fourier Transform
MTI	Moving Target Indicator
FCNs	Fully Convolutional Neural Networks
ResNet	Residual Network
SAR	Synthetic Aperture Radar
